# Dynamic Mortality Risk Prediction in Myelodysplastic Syndromes Using Longitudinal Clinical Data

**DOI:** 10.1200/CCI-25-00236

**Published:** 2025-12-23

**Authors:** Jonathan Bobak, Philipp Spohr, Sarah Richter, Alexander Streuer, Felicitas Isabel Schulz, Corinna Strupp, Catharina Gerhards, Nanni Schmitt, Thomas Luft, Sascha Dietrich, Ulrich Germing, Gunnar W. Klau

**Affiliations:** ^1^ Department of Hematology, Oncology and Clinical Immunology, Medical Faculty and University Hospital Düsseldorf, Heinrich Heine University Düsseldorf, Düsseldorf, Germany; ^2^ Chair Algorithmic Bioinformatics, Heinrich Heine University Düsseldorf, Düsseldorf, Germany; ^3^ Center for Digital Medicine, Heinrich Heine University Düsseldorf, Düsseldorf, Germany; ^4^ Department of Medicine, Hematology, Oncology and Rheumatology, University Hospital, Heidelberg, Germany; ^5^ Department of Hematology and Oncology, Medical Faculty Mannheim, Heidelberg University, Mannheim, Germany; ^6^ Institute for Clinical Chemistry, Medical Faculty Mannheim of the University of Heidelberg, Mannheim, Germany

## Abstract

**PURPOSE:**

Patients with myelodysplastic syndromes (MDS) exhibit diverse disease trajectories necessitating different clinical approaches ranging from watch-and-wait strategies to hematopoietic stem cell transplantation. Existing risk scores like the IPSS-R or Endothelial Activation and Stress Index provide static risk stratification at diagnosis but do not capture evolving disease dynamics. We addressed this problem by introducing a dynamic, data-driven approach to repeatedly predict short-term mortality risks, across the patient's disease course.

**MATERIALS AND METHODS:**

We developed a machine learning model on the basis of gradient-boosted decision trees to estimate 1-year mortality risks from both longitudinal parameters from blood values and diagnosis-based features. We trained the model on a data set of patients from the MDS Registry Düsseldorf (n = 1,024) and validated it on patients from University Hospitals Heidelberg (n = 286) and Mannheim (n = 31).

**RESULTS:**

Validations on independent cohorts achieved area under the receiver operating characteristic curve scores of around 0.8 and better predictive performance for 1-year mortality compared with a diagnosis-only baseline model. The model accurately predicted mortality risks as early as within the first 90 days of diagnosis. Feature importance analysis revealed clinically plausible feature-label relations, supporting interpretability. Comparison with the IPSS-R and training on 1-year AML progression revealed the advantages and generalizability of the approach.

**CONCLUSION:**

This dynamic risk model enables continuous, individualized assessment of 1-year mortality risk in patients with MDS, offering a supplement to static scores used at diagnosis. Our results highlight the utility and importance of including longitudinal parameters in risk assessment analysis.

## INTRODUCTION

Myelodysplastic syndromes (MDS) are a class of malignant stem cell diseases resulting in reduced and defective blood cells. The main affected age group are elderly patients, with survival times ranging from a few months to multiple years.^1^ Quantitative mortality risk assessment for this diverse set of clinical trajectories is paramount in clinical decision making. The main approach for MDS in recent years has been to focus on diagnosis-based modeling. Scores like the International Prognostic Scoring System are broadly applied in the form of the revised and molecular IPSS-R/IPSS-M^2,3^ as well as other scores^4,5^ relying on the same methodology. An advantage is the simplicity of calculation. Only five parameters are needed to classify a patient into one of five IPSS-R categories ranging from very low to very high risk. However, this simplicity and focus on a single point in time leads to losses in predictive power over time, especially for lower-risk categories.^6^ Additionally, the categorization effectively limits predictions to five hazard profiles and does not allow for individualized predictions as patients may not follow the assigned risk profile.

**CONTEXT**

**Key Objectives**
How can we leverage longitudinal data and machine learning to improve dynamic mortality risk assessment for patients with myelodysplastic syndromes?
**Knowledge Generated**
A new approach to dynamic longitudinal risk assessment on the basis of gradient boosted decision trees was formulated using features from both diagnosis and blood tests on follow-ups to predict 1-year mortality risks. The approach allows for repeated predictions, is generalizable to multiple cohorts and labels, and performs better than diagnosis-related scores.
**Relevance *(J.L. Warner)***
This study suggests a modern and informative approach to early risk assessment in MDS, with external validation. It would be interesting to see if similar methods could extend risk prediction even further than 1-year mortality, in the future.*
*Relevance section written by *JCO Clinical Cancer Informatics* Editor-in-Chief Jeremy L. Warner, MD, MS, FAMIA, FASCO.

For longitudinal predictions, clinicians must rely on their experience since re-evaluation of diagnosis-based scores is not always possible and may require more intensive diagnostics rarely performed on follow-up. The WHO-based prognostic scoring system^7^ investigated a time-adjusted score with good predictive performance for overall survival and AML progression but is still limited to five risk categories. Longitudinal data also come with lots of challenges like unevenly sampled or missing data and more complex patterns.

To address these challenges, prediction tools based on machine learning algorithms are a major focus to recognize patterns and improve predictions. Mosquera Orgueira et al^8^ applied random survival forests to improve model performance compared with the IPSS-R showing promising improvements for individualized predictions and use of nonlinear models to learn patterns. Tree boosting was also used to predict MDS diagnosis risks 1 year before clinical diagnosis with good classification performance.^9^ Apart from single point in time predictions, dynamic predictions have been applied to analyze 100-day mortality risks after hematopoietic stem cell transplantation. Risks are predicted each day starting on the day of transplantation up until day 30 showing that with increasing longitudinal data predictions improved.^10^

**FIG 1.**
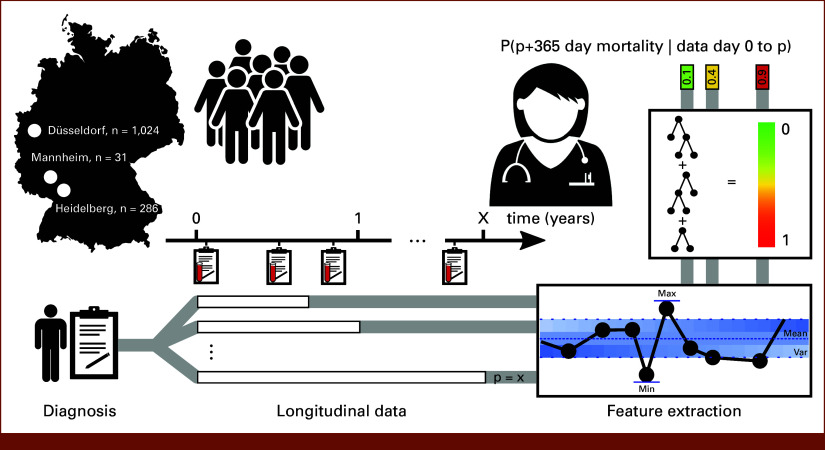
Overview of our study pipeline. We use the Düsseldorf patient data set for training and initial validation of our method supplemented by two validation data sets from the University Hospitals Heidelberg and Mannheim. For each patient, we gather both diagnosis-based and longitudinal features. For the retrospective longitudinal data, we subsample each patient at different time points and extract a standardized set of features. Combined with diagnosis-based features, these serve as input for a gradient-boosting model which predicts a probability between 0 and 1 (colored bar) for 1-year mortality for each subsample. A higher predicted probability is interpreted as a higher risk of dying within 365 days after the time of prediction.

Here, we present a dynamic approach leveraging both diagnosis-based and longitudinal features. We trained and validated the model on retrospective data from the MDS Registry Düsseldorf and the University Hospitals Heidelberg and Mannheim showing promising prediction improvements by using longitudinal data as model input. A conceptual overview of our approach is shown in Figure 1.

## MATERIALS AND METHODS

### Patient Inclusion Criteria

Our inclusion criteria for patients, related to necessary minimal data availability and bias reduction, included the following:

1. Surviving at least 6 months,
2. Receiving no hematopoietic stem cell transplantation (HSCT) at any time, as this procedure is heavily disease altering,
3. Having at least three values for each longitudinal variable,
4. Having at least 1 year of follow-up for right-censored patients.

By setting a minimal survival time, we aimed to reduce leading bias for very high-risk patients diagnosed very late in disease progression. This does not fully eliminate the bias but allows us to focus on more subtle, long-term disease patterns.

### Conceptual Framework

We follow the framework described by Sherman et al,^11^ defining a fixed reference point $p_{0}$ as the time of diagnosis. Each prediction $p_{i}$ at time i incorporates all available data between $p_{0}$ and $p_{i}$, estimating the probability of death within 365 days from i.

This setup ensures that predictions are temporally localized, incorporate full clinical history up to time i, and are independent of prior predictions and the outcome itself. Although prediction windows may overlap, each instance is treated independently. Successive predictions advance the prediction horizon.

### Data Preprocessing and Feature Extraction

Patient data have two modalities. Diagnosis-based features were only observed at $p_{0}$ while longitudinal features were measured repeatedly. Longitudinal information is irregular and sparse as patients are monitored in a real-life clinical environment.

Each patient's complete retrospective longitudinal data were subsampled each quarter with observations. Quarterly sampling was chosen such that densely observed hospitalization periods are not overrepresented, but a representative set of intermediate prediction points is obtained. The point $p_{i}$ is the last observation within a quarter. Owing to the decreasing number of data points, sampling was stopped after 32 quarters.

To prevent label leakage and train on more subtle signals, we excluded the final 60 days before death from sampling.

From the irregular and sparse longitudinal data, we extracted a standardized set of characteristic features for each subsample (Appendix Table A1). These derived features were then combined with the diagnosis-based features to construct the final input vector.

Samples were labeled positive (1) if death occurs 365 days after $p_{i}$ or negative (0) otherwise. A patient may have samples with different labels which are obtained independently of one another.

### Model Architecture

Our longitudinal survival prediction model using XGBoost^12^ incorporates both diagnosis-based and longitudinal features. The input features used for model training are summarized in Table 1. The longitudinal features were mainly chosen because of their frequent observation in the clinical setting without bias toward adverse events. Bone marrow blasts and cytogenetics were only included at diagnosis as follow-ups to these variables are biased toward clinical deterioration and disease progression. We excluded the IPSS-R^2^ from the feature set since most of its constituent variables were already model inputs. We did not consider therapies as inputs because of uncertainty around dates and a huge variety of treatment schemes. The model has to learn related patterns implicitly allowing it to cope better with new therapies since the input features do not depend on them. Mutational data could not be incorporated because of sparse and incomplete data.

**TABLE 1.** Input Features for Model Training

Diagnosis-Based Features	Longitudinal Features
Age	Hemoglobin (g/dL)^a^
Sex	Leukocytes (×1,000/μL)^a^
Bone marrow blasts, %^b^	Erythrocytes (Mio/μL)
IPSS-R Karyotype classification^2;^^b^	Hematocrit, %
EASIX (baseline only)^13;^^b^	Thrombocytes (×1,000/μL)
	Time series length (in days and quarters)

NOTE. Diagnosis-based parameters have singular values while the longitudinal features correspond to series of observations. EASIX is just used for the baseline model. The survival time is input for both models and is given in fine-grained days as well as 90-day quarters.

Abbreviation: EASIX, Endothelial Activation and Stress Index.

a Both hemoglobin and leukocytes are present in the baseline and longitudinal models. For the baseline model the measurements at diagnosis and for the longitudinal model a series of measurements is used.

b These features are optional, that is, the models can handle missing values for them, at least one feature is required.

As a comparative baseline, we trained a model only on diagnosis-based features. Contrary to the longitudinal model, we included the Endothelial Activation and Stress Index score^13^ as a replacement for raw thrombocyte counts.

The XGBoost gradient boosting implementation natively handles missing data by adding additional decision branches, when a value is missing, allowing us to retain patients with missing diagnosis-based features. Hyperparameters were selected via grid search with cross-validation on the training set. To reduce overfitting given the modest data set size, we applied regularization strategies and minimized false negatives by tuning model sensitivity with sample weights during training (Appendix Table A2).

### Evaluation Methodology

Classification performance was evaluated using three metrics: area under the receiver operating characteristic curve (AUROC^14^), area under the precision-recall curve (AUPRC^15^), and Brier^16^ Score. The AUPRC is particularly relevant because of the observed class imbalance, which the AUROC metric has been shown to omit.^15,17^ The Brier Score quantifies the absolute error in relation to the true probabilities.

For a robust initial model performance assessment, we conducted repeated cross-validation on the Düsseldorf training cohort. Metrics were averaged over all cross-validation folds to account for variability because of cohort composition and to assess general model capability. Changes in performance for predictions further from diagnosis were evaluated by calculating metrics within each quarterly bin.

Model performance was compared with the established IPSS-R^2^ using the same cross validation strategy (Appendix, Comparison to the IPSS-R section). A comparison with the more recent IPSS-M^3^ was not possible since too little genomic information was available.

To evaluate generalizability across institutions and real-world applicability, we trained our model on the Düsseldorf data set and evaluated it on two independent validation cohorts from University Hospitals Heidelberg and Mannheim.

Finally, we performed feature importance analysis on this generalizable model using the average reduction in impurity achieved for each feature over all trees, and shapely additive explanations (SHAP) values^18^ a game theoretic approach to explain relations between input features and model outputs.

### Data Sets

The training cohort consisted of 1,024 retrospective patients with MDS treated at the University Hospital Düsseldorf, all part of the Düsseldorf MDS Registry, with 6,146 samples and a 2.66:1 ratio between negative and positive labels. We validated on two retrospective cohorts from University Hospitals Heidelberg (patients = 286, samples = 1,708, label balance ≈ 4:1) and Mannheim (patients = 31, samples = 237, label balance ≈ 10:1). For the validation sets, we did not filter the final 60 days of longitudinal data to simulate real predictions without event knowledge.

**FIG A1.**
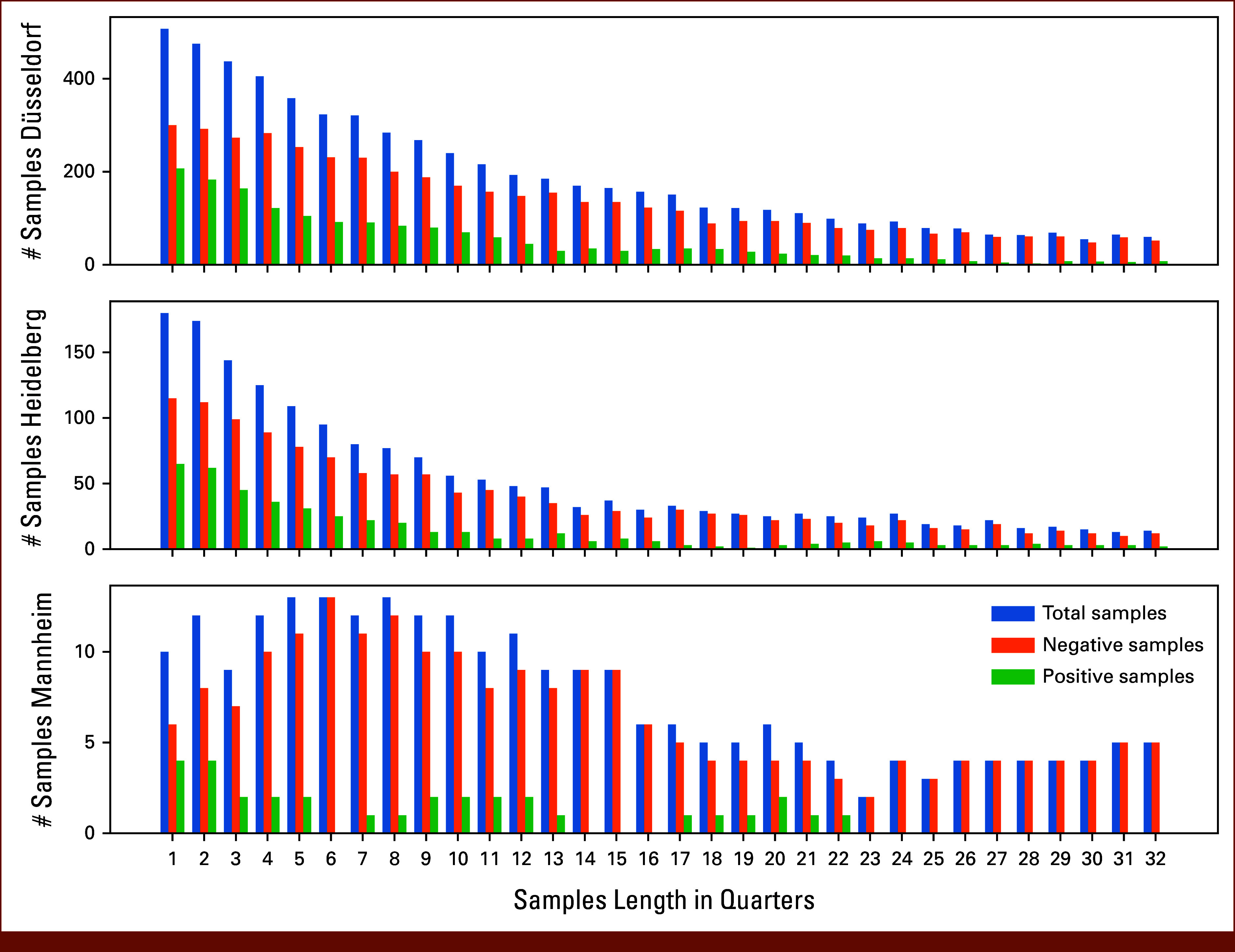
Sampling lengths over time given for each quarter. Negative samples, meaning the patient did survive at least 365 days after the time of prediction, have label 0 while positive ones, where the patient died within 365 days, have label 1.

Sample numbers diminished over time with a similar decrease across data sets and labels (Appendix Fig A1). The Heidelberg and Düsseldorf cohorts show a lower median follow-up and higher mortality than the Mannheim data set indicating more high-risk patients with earlier events but similar average sample numbers per patient. Detailed demographics and characteristics can be found in Appendix Table A3.

### Software Availability

The pipelines used to train and evaluate the model were written in python using snakemake.^19^We used tsflex^20^ and tsfresh^21^ for feature extraction; XGBoost,^12^ SHAP^18^ and scikit-learn^22^ for model training and evaluation; Pandas^23,24^ and NumPy^25^ to handle and transform data; and matplotlib^26^ for plotting. All code is available in GitHub.^27^ A small webtool, to test both models, is available in Research Group Dietrich.^28^

## RESULTS AND DISCUSSION

### Adding Longitudinal Data Improves Predictions

Table 2 lists results for all metrics and data sets for the comparison between longitudinal and baseline models. On the Heidelberg data set, the longitudinal model improves the AUROC by approximately 0.11 and the AUPRC by 0.21. Both models perform substantially better than random guessing, which would yield an AUROC of 0.5 and an expected AUPRC of around 0.2. Furthermore, the longitudinal model exhibits a lower Brier score than the baseline, indicating improved overall calibration and probabilistic accuracy.

**TABLE 2.** AUROC, AUPRC, and Brier Score for All Three Data Sets

Data Set	Metric	Baseline	Longitudinal	Difference
Düsseldorf cohort (cross-validation mean)	AUROC	0.6841	**0.8086**	+0.1245
	AUPRC	0.4600	**0.6187**	+0.1587
	Brier score	0.2147	**0.1691**	–0.0456
Mannheim cohort	AUROC	0.6391	**0.8552**	+0.2161
	AUPRC	0.2757	**0.5820**	+0.3063
	Brier score	0.2022	**0.1986**	–0.0036
Heidelberg cohort	AUROC	0.7232	**0.8364**	+0.1123
	AUPRC	0.4873	**0.7057**	+0.2184
	Brier score	0.2035	**0.1519**	–0.0516

NOTE. For the Düsseldorf cohort, averages from cross validation are given. For the Heidelberg and Mannheim data sets, performance is measured on models trained on all patients from Düsseldorf. Results are rounded to four decimal places. For AUROC and AUPRC higher values, for the Brier Score lower values are better. Bold entries denote best performing model for the respective metric and dataset.

Abbreviations: AUPRC, area under the precision-recall curve; AUROC, area under the receiver operating characteristic curve.

For the Mannheim cohort, similar improvements for the longitudinal model can be seen for the AUROC and AUPRC. Interestingly, Brier scores for this data set do not show the same difference but are close across models.

The baseline model performs as well as the IPSS-R with the advantage of being able to cope with missing values shown in Appendix (Comparison to the IPSS-R section). The longitudinal model outperforms both other models. All subsequent comparisons were performed between the baseline and longitudinal model as they allow for larger patient cohorts.

The intuitive assumption, that adding information from longitudinal data improves model performance compared with an approach solely reliant on data from the time of diagnosis, is confirmed. We conclude that our longitudinal model trained on patients from Düsseldorf learns generalizable patterns and is applicable to other, real-world cohorts.

### Longitudinal Model Can React to Dynamic Changes

**FIG 2.**
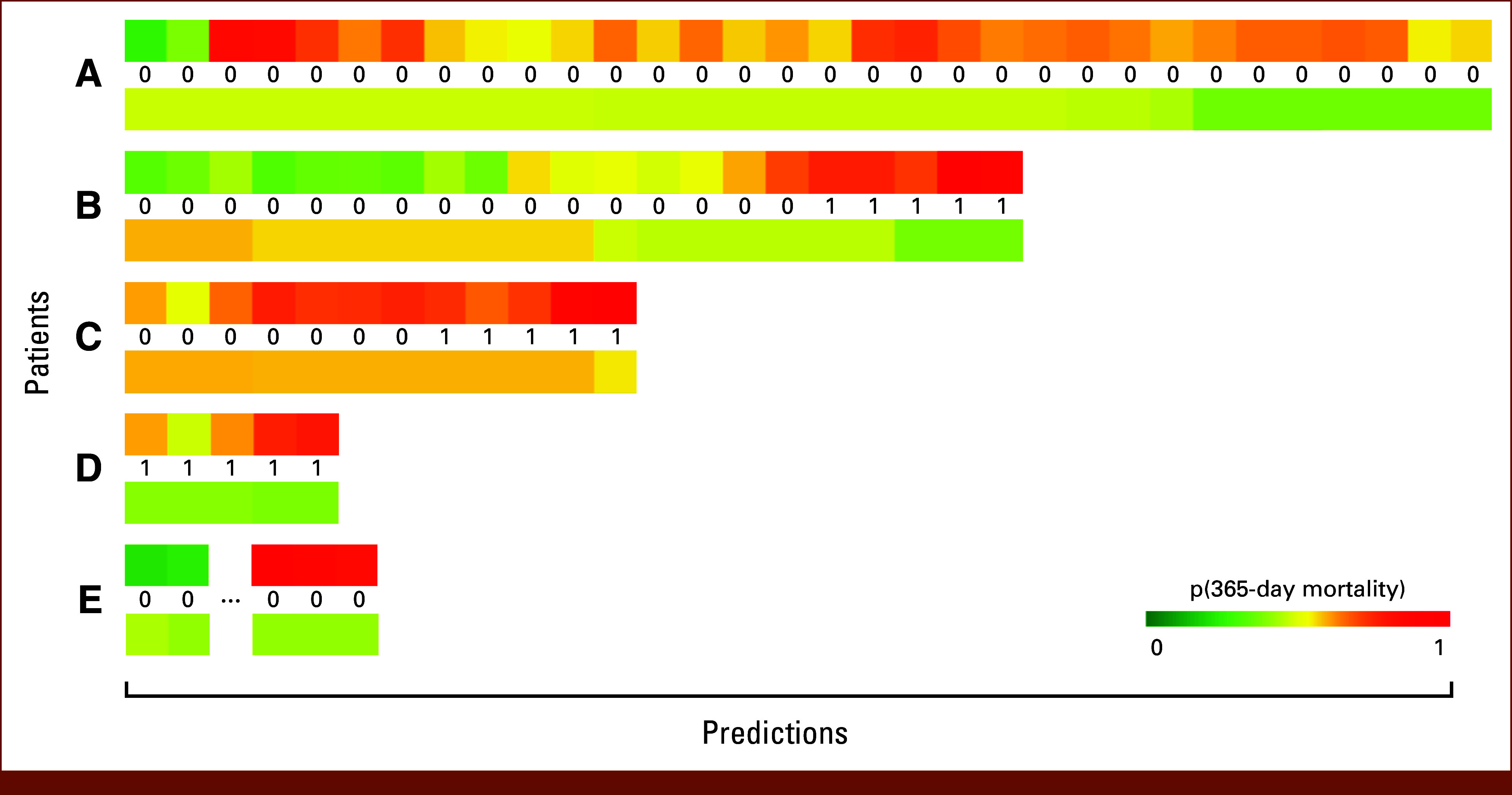
Example predictions for five different patients. Each block in the figure represents a prediction for a sampled quarter. The numbers between the colored bars correspond to the true label for each prediction; the top bar displays the probabilities given by the longitudinal model, and the bottom bar those given by the baseline model. Green is associated with likely survival, whereas red indicates a high risk for dying 365 days after the time of prediction. For patient D, we were able to sample the longitudinal data five times in 90 day (quarterly) intervals. All these samples were assigned the positive label, since the patient died within 365 days of the prediction time (middle). The longitudinal model (top) predicted higher risks for all samples compared with the baseline model (bottom). Patient E has two samples right after diagnosis, but then a longer period of missing values before we were able to sample again when the patient was readmitted because of a progression to AML.

We observe differences in the two models' approaches to predicting risks. The baseline model predicts based on the initial state of a patient and their survival time. The correlation between survival time and mortality drives estimation and is very similar to classical survival analysis in the form of Cox regression or Kaplan-Meier curves.^29,30^ Predicted risks of different subsamples in one patient generally decrease monotonically over time. Figure 2 shows example patients with predictions from both models as well as the ground truth labels. Patients A and B start with gradually decreasing risks. For patient A, this is correct, as there are no positive samples. Patient B died shortly after the 21st quarter, but the baseline model predicts a low risk. For patient D, the predictions indicate low risk from the start although the patient only has positive samples.

The longitudinal model predicts dynamically as it incorporates new and changing longitudinal data. For patient B, we observe an increasing risk over time with the highest mortality probabilities shortly before the event. A similar pattern is observed for patients C and D including a higher initial risk for patient D compared with the baseline model.

Dynamic predictions from longitudinal features are advantageous while the baseline model is bound to survival time and population dynamics resulting in probabilities being independent of a patient's actual disease trajectory similar to classical survival curves.

### Learning Generalizable Patterns and Training on Other Labels

For the two external validation sets, we explored how the longitudinal model predicted samples within the last 60 days before the occurrence of a mortality event as they would not be excluded from training. For all samples falling under this condition, the longitudinal model predicted either higher or equal mortality risks compared with prior predictions. Both patients B and C in Figure 2 died within the last quarter of predictions, and all predictions in the 60-day interval show the highest predicted risk. We can see that the patterns of longitudinal features right before the mortality event are either similar to, more pronounced or continuations of patterns from earlier prediction points.

One-year mortality risk is only one possible label for training. To investigate if our pipeline is adjustable to other labels, we also trained on 1-year AML progression risk, a common adverse event, and observed similar performance advantages for the longitudinal model over the baseline model and scores like the IPSS-R (see Appendix, Comparison to the IPSS-R and AML Progression as a Prediction Label sections). This observation underscores the potential of including longitudinal data into risk assessment models and highlights the generalizability of our approach.

Additionally, we qualitatively investigated whether events like AML progression and transfusion burden were reflected in predictions of our initial model trained on the 1-year mortality label. Patient A in Figure 2 received transfusions over the entire observation period and was screened for a study investigating iron overload. This may explain why the longitudinal model predicts a constant risk for this patient, as 8 years of close clinical monitoring and regular transfusions do not indicate a low-risk trajectory. For AML progression, the model reported higher mortality risks around the time of recorded progression for most patients in both validation cohorts. Patient E progressed to AML shortly before the third prediction, which shows an increased mortality probability although the patient survived until the end of our observational period, indicating a higher risk trajectory after progression.

Although the model seems to associate higher risk with specific events, it is not conclusive whether the predictions are based on causal factors or their effects, as clinical interventions may already begin before progression is clinically observed or transfusions are administered.

### Trade-Off Between False Positives Versus False Negatives

As seen exemplary for patient A in Figure 2, the longitudinal model is prone to false-positive predictions. One reason may be medical aspects related to therapy and disease progression. Another influencing factor was our choice to introduce sample weights during training, increasing the penalty for false-negative predictions and pushing the model toward a high recall for positive samples. We qualitatively investigated false negatives in both validation data sets. Many of them could be attributed to short-term mortality or events unrelated to MDS. Some examples include septic shock after hip surgery or liver failure because of liver cancer. Although MDS is a contributing comorbidity, our model cannot predict these events, if the resulting cause of death is not directly visible within the longitudinal data. This may occur if the patient was treated elsewhere, the blood values were not available, or the cause of death does not reflect within the blood values.

### Model Performance Does Not Depend on Sample Length

**FIG 3.**
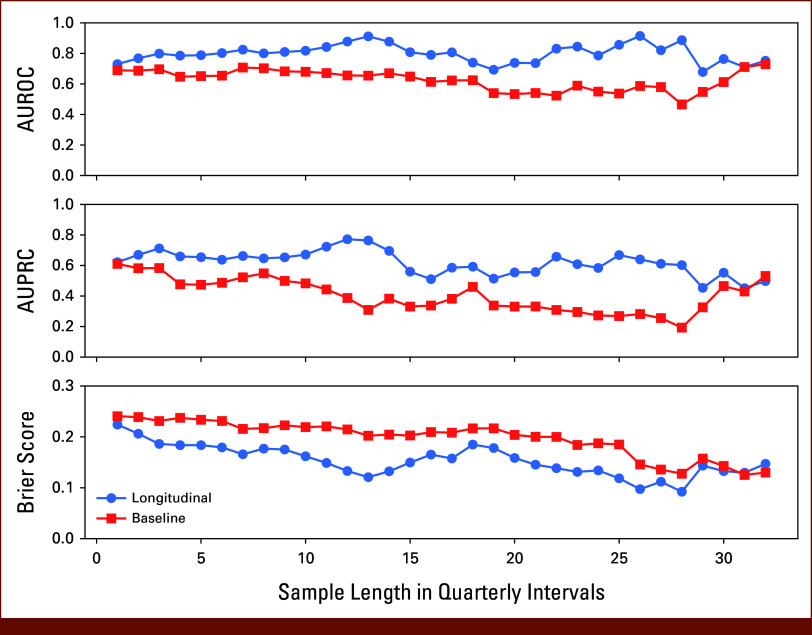
Average evaluation metric values when performing cross-validation on the Düsseldorf data set. For AUROC and AUPRC higher, while for the Brier Score, lower values are better. See Appendix Figure A2 for information on variance across cross-validation runs. Increasing variance and reduced sample size are reasons for both models converging to similar performance. AUPRC, area under the precision-recall curve; AUROC, area under the receiver operating characteristic curve.

To investigate predictive power over time, we performed cross-validation on the Düsseldorf data set, tracking average evaluation metrics for each split and sample length. All metrics show a similar trend (see Fig 3). The difference between the longitudinal and baseline models starts out small but increases over the first quarters with the longitudinal model outperforming the baseline. Overall, the longitudinal model stabilizes around an AUROC of 0.8 and an AUPRC of 0.6 with improving Brier scores but becomes more variable for longer sampling periods. The baseline model shows a decreasing performance trend for both AUROC and AUPRC with a constant Brier score apart from the last 4-5 quarters with an increasing performance trend.

**FIG A2.**
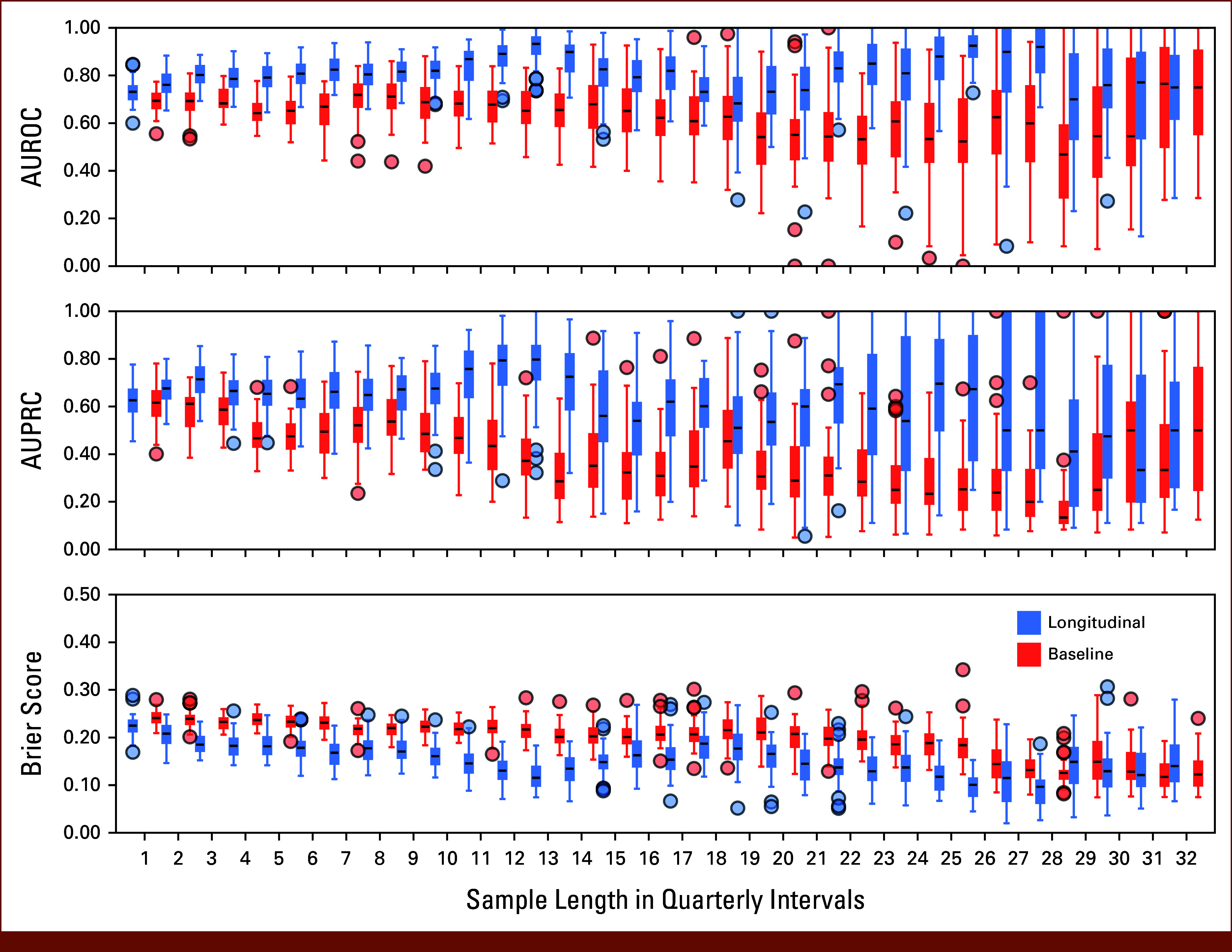
AUROC, AUPRC and Brier Score with variance bars on the cross-validation run on the Düsseldorf data set. Bars represent IQR with the whiskers extending to 1.5 × IQR. Outliers are denoted by circles. AUPRC, area under the precision-recall curve; AUROC, area under the receiver operating characteristic curve.

Already without extensive longitudinal data, the longitudinal model performs better than the baseline. We cannot completely rule out data set specificities as reasons for the performance trends but the differences in model performance are persistent, and we observe independent trends between models across cross-validation folds leads us to conclude that the longitudinal model learns generalizable trends. One caveat is the high variance across splits (Appendix Fig A2) due to diminishing amounts of samples and changes in label balance. However, this variance is consistent for both models supporting observations on average performance.

The early advantage of the longitudinal model indicates that disease dynamics hold predictive value early on, stressing the importance of continued quantitative risk monitoring.

### Meaningful Feature—Label Relations

**FIG 4.**
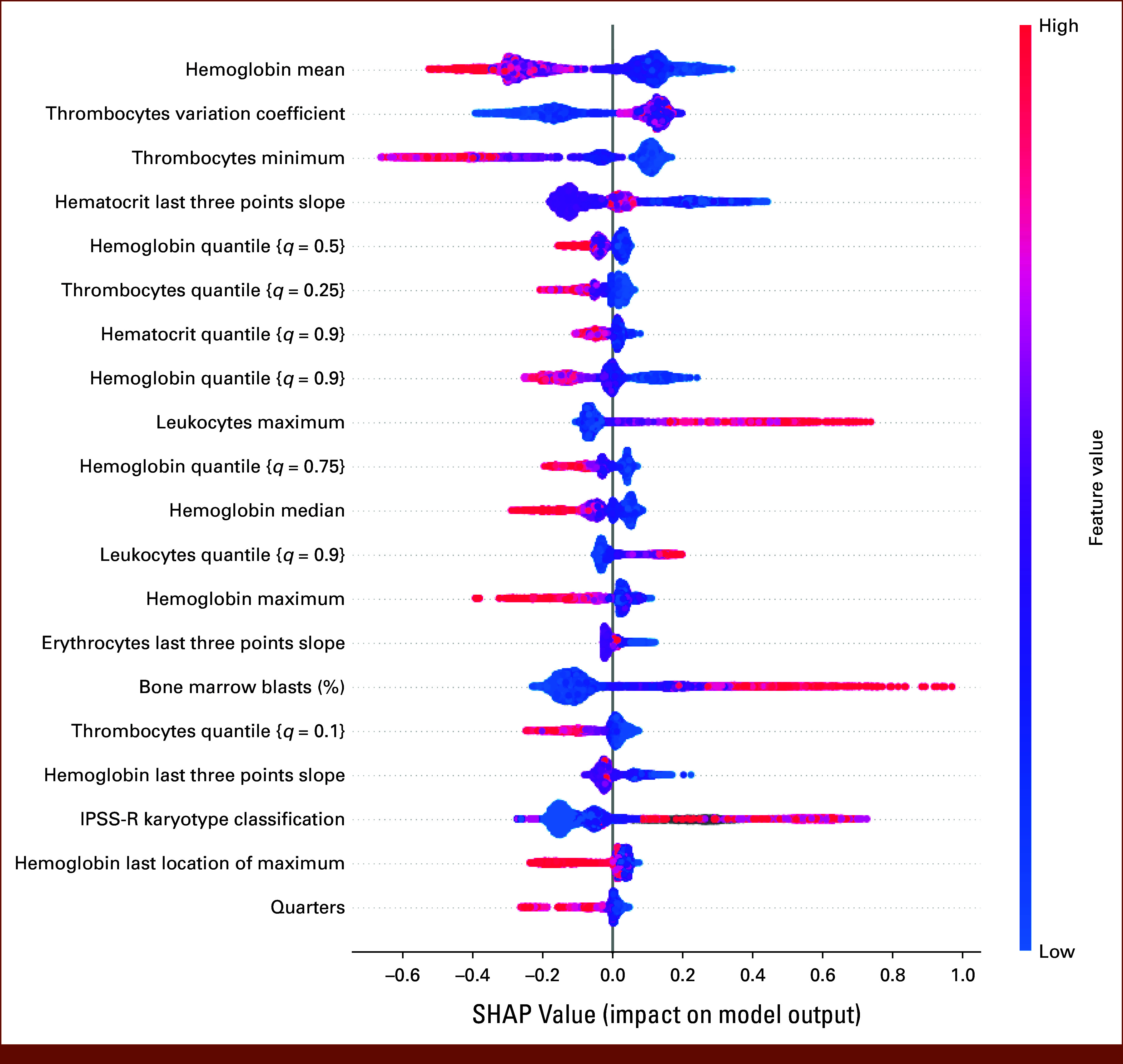
SHAP values for the 20 most important features according to the XGBoost gain metric. Gain describes the average reduction in loss when a feature was chosen in a split across all trees and training samples. For each feature and sample, the SHAP value gives an intuition if the feature biases the prediction toward the positive or negative label. Over all samples this results in the shown density distinguishing higher and lower feature values by color. SHAP, shapely additive explanations.

Feature importance evaluations show meaningful feature-label relations validated by medical domain experts for the 20 most important features as seen in Figure 4. Exemplary, lower hemoglobin averages are associated with increased mortality risks. According to the MDS treatment strategy, patients with lower hemoglobin values require transfusions,^31^ and hemoglobin has been a robust marker for risk assessment.^2,3^ We observe 7 of the 20 most important features relate to quantile distributions. This may be indicative of the model learning to detect outliers. Supporting this hypothesis is the inclusion of variation coefficients and averages in this list. One reason may be that patients who regularly require transfusions will likely exhibit more variance and present with outliers. There has been evidence that increased transfusion-need is correlated with worse outcome.^5,32^ Another feature type we observe is the slope of the last three data points of longitudinal variables. It captures a more localized view of a patient's recent history and short-term changes.

### Clinical Implementation

The clinical application of the longitudinal model, pending further validation, would supplement existing risk scores like the IPSS-R or IPSS-M. Our approach does not allow for predictions from the day of diagnosis but improves risk stratification after the first few follow-ups. The individualized and dynamic predictions can be directly used as a warning system for the target label. From a technical standpoint, the longitudinal model does require more input than traditional approaches; however, all code and format specifications are readily available for adaptation to local IT infrastructures.

In conclusion, we present a new approach to quantify dynamic 1-year mortality risk in patients with MDS using gradient boosted decision tree ensembles. On the basis of two validation cohorts and cross-validation on the training set, we can show the advantages of using longitudinal features outperforming a purely diagnosis-based model with improvements among all evaluated scores. Longitudinal data add important information to the prediction from early on. Additionally, we can show that the model does generalize to other, independent data sets, while learning meaningful label-feature relations correlating to known medical factors and disease markers. Even beyond the learned label, the model often reports higher risks on adverse events like AML progression and transfusion burden. We also show that the model can be trained on different labels like 1-year AML progression with good classification performance compared with existing scores and is not limited to 1-year mortality.

Even with three university clinics participating, the number of samples limits the interpretability, and our results should be seen as a proof of concept. For a fully validated and clinically usable model, more data from additional centers across multiple countries would be needed. The lack of molecular information remains a limitation because of its sparse availability as well. Newer risk scores like the IPSS-M do show improved predictions with molecular information at diagnosis but are still not as commonly applied in the clinical routine as the IPSS-R. However, we do see potential for our model by using molecular data as input.

The model is applicable in different environments as shown by the federated validation on the Heidelberg data set.

We hope to further validate and improve the approach looking at other clinical labels of interest like therapy onset. Owing to the dynamic and easily adjustable pipeline, other features both at diagnosis and longitudinal can be tested for predictive power.

Inclusion of both machine learning and longitudinal features into the clinical routine to support decision making and analyze large amounts of personalized data is crucial to improve patient care and survival.

## PREPRINT VERSION

Preprint version available on medRxiv (https://www.medrxiv.org/content/10.1101/2025.07.21.25331775v1).

## Figures and Tables

**FIG A3.**
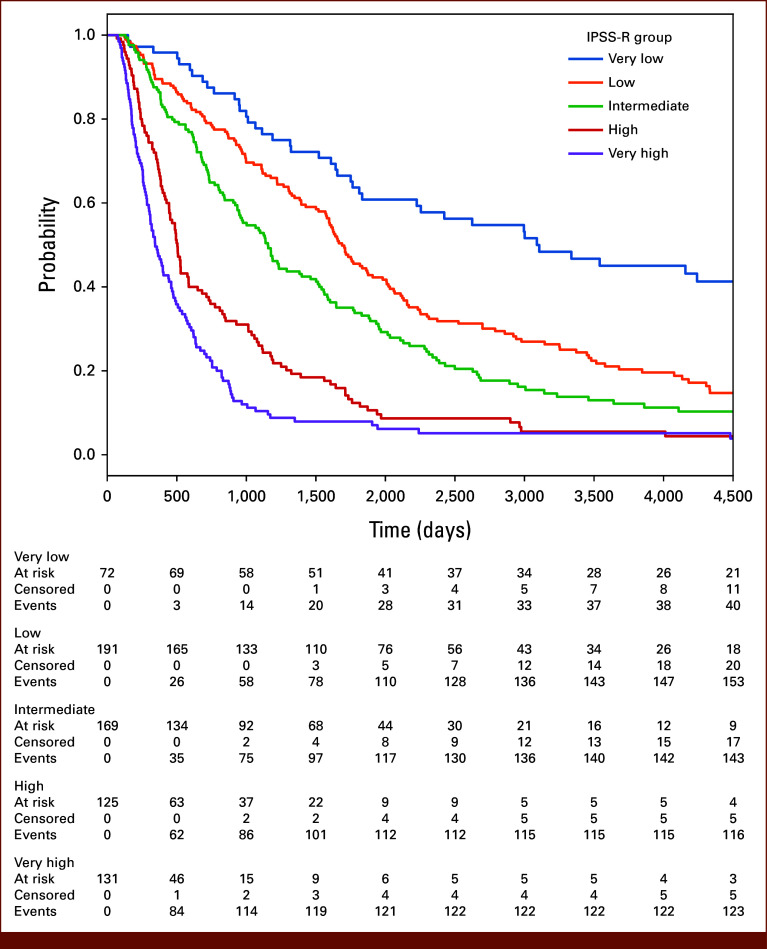
Kaplan-Meier curves for the 1-year mortality cohort of n = 688 patients and all IPSS-R risk categories. We see the expected differentiation from very-high to very-low risk categories and similar median survival times as reported by Greenberg et al.^2^

**FIG A4.**
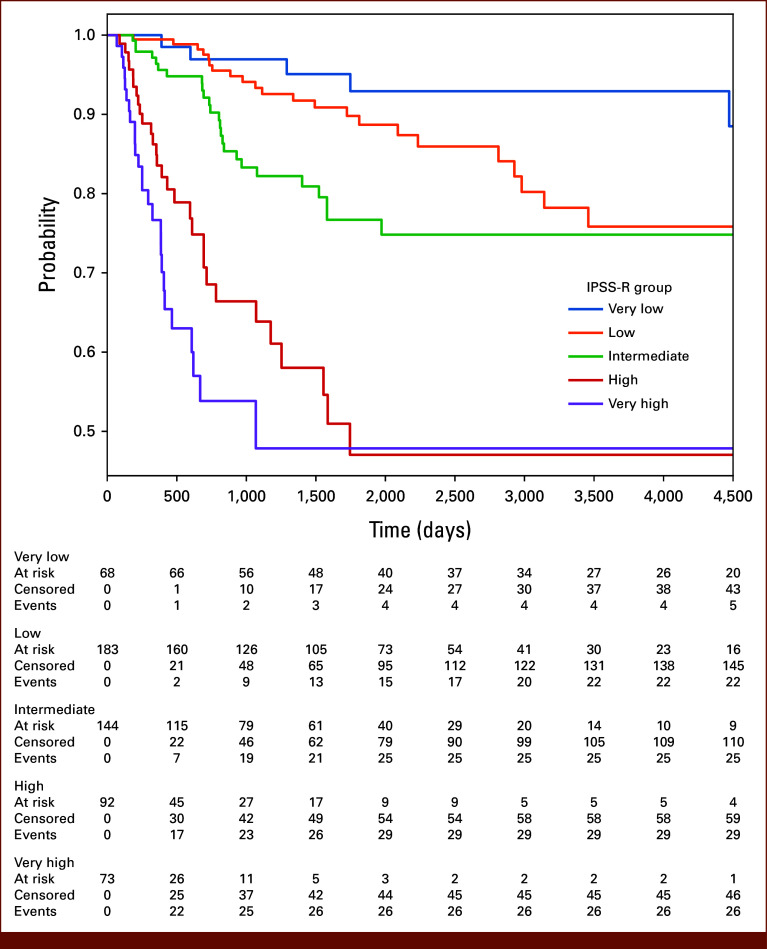
Kaplan-Meier curves for the AML progression cohort of n = 431 patients and all IPSS-R risk categories. The event used was AML progression. We see the expected differentiation from very-high to very-low risk categories similar to results reported by Greenberg et al.^2^

**FIG A5.**
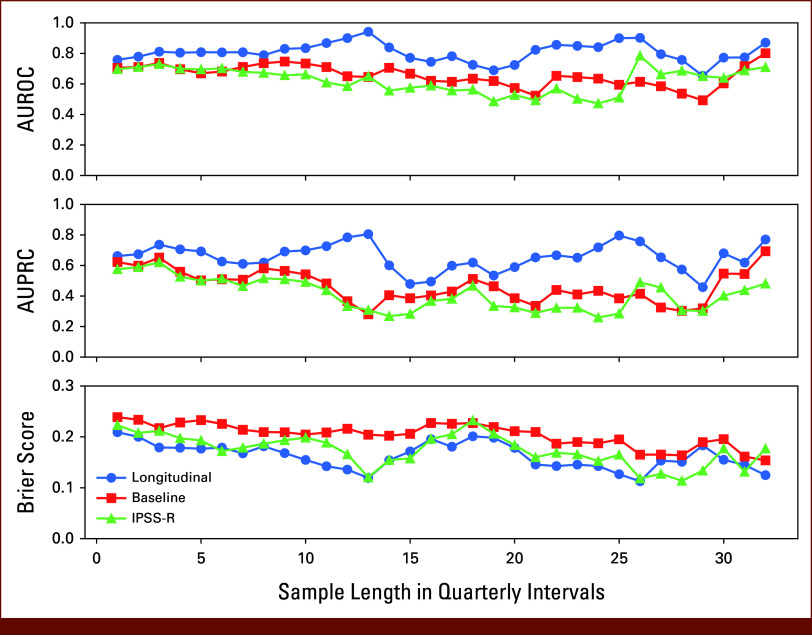
Comparison of average evaluation metrics over time between IPSS-R, on the 1-year mortality label, and the baseline and longitudinal models. The data set is based on all samples from the Düsseldorf data set for patients with a present IPSS-R. This limits the data set to n = 688 patients.

**FIG A6.**
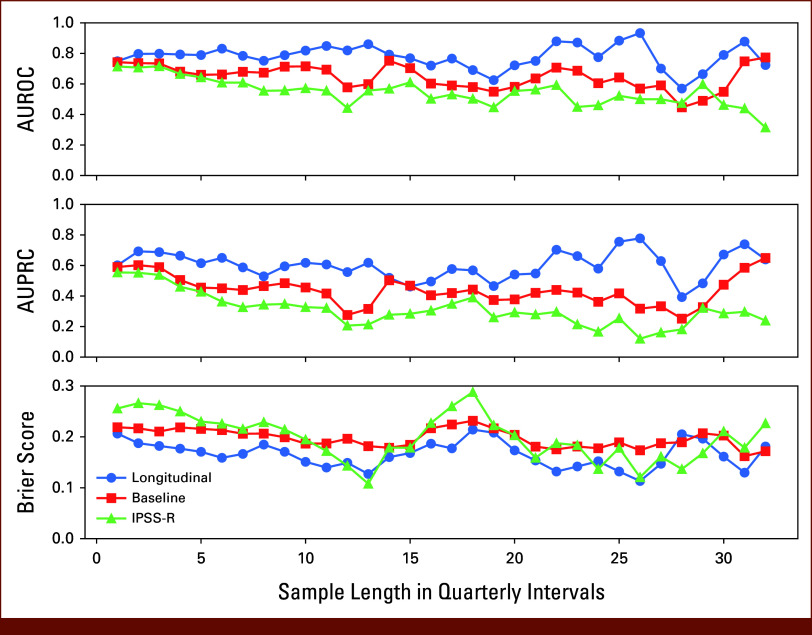
Comparison of average evaluation metrics over time between IPSS-R, on the 1-year AML progression label, and the baseline and longitudinal models. The data set is based on all samples from the Düsseldorf data set for patients with a present IPSS-R. This limits the data set to n = 481 patients.

**FIG A7.**
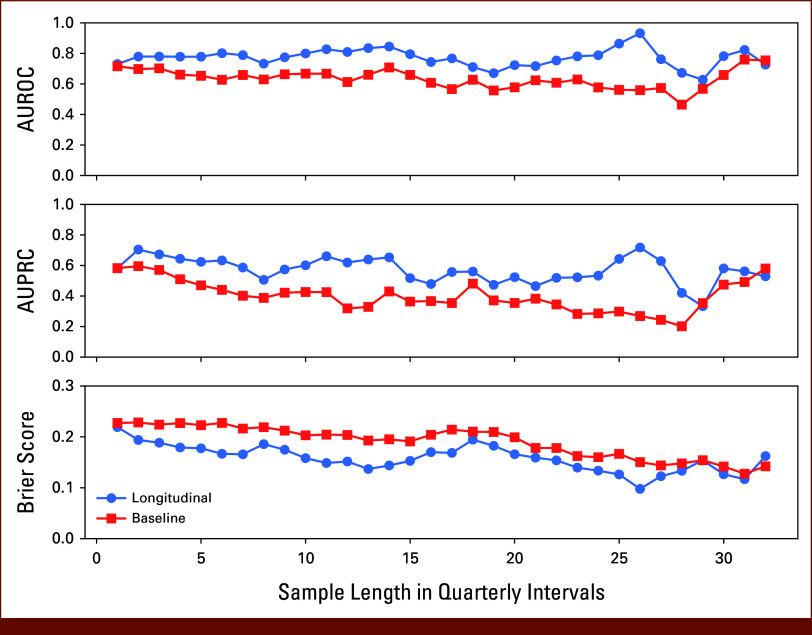
Comparison of average evaluation metrics over time between IPSS-R, on the 1-year AML progression label, and the baseline and longitudinal models. The data set is based on all samples from the Düsseldorf data set for patients with a present IPSS-R. This limits the data set to n = 481 patients.

## APPENDIX

### Comparison With the IPSS-R

To validate that our baseline model is a good representation of a diagnosis-based predictor, we compared both models to the IPSS-R. As bone marrow blast count and karyotype were not regularly observed on follow-up without a bias toward clinical events like AML progression, we decided to compute the IPSS-R once at diagnosis and leverage conditional Kaplan-Meier curves to assign 1-year mortality probabilities to each sample.^30^

We also used the AML progression date to train our model for 1-year AML progression risk as the IPSS-R was also evaluated on it.

All inclusion criteria remained the same for both labels but the IPSS-R was required to be present. As we recalculated all age-adjusted IPSS-R scores, this also implies the presence of bone marrow blast percentages and karyotype classifications at diagnosis.

For the 1-year AML progression label, some patients were filtered because of the inclusion criteria conflicting with the changed event time.

Specific patient and sample numbers are given in Table A4. To ensure that the data sets are representative in terms of IPSS-R category distribution, we plotted the respective Kaplan-Meier curves for both labels in Figures A3 and A4.

The comparison was performed using repeated cross-validation on the Düsseldorf data set, resulting in 50 train/test splits.

We first evaluated the 1-year mortality label. As seen in Table A5, the baseline has a small advantage over the IPSS-R model in terms of AUROC and AUPRC, whereas the IPSS-R has a smaller Brier Score. Both models are outperformed by the longitudinal model with margins of 0.1 to 0.15 in terms of AUPRC and AUROC and a slightly lower Brier Score compared with the IPSS-R.

We see the same trend on the 1-year AML progression label, although with smaller margins between models. Lower absolute values, although not directly comparable between labels as different data sets were used, may indicate that the overall predictive quality decreases. One reason could be the larger label imbalance in the AML data set. However, we were not able to combat the performance drop by modifying sample weights during training or changing other hyperparameters. This points to the label itself being more complex to predict. In combination with the observation that training on the 1-year mortality label reflected AML progression events as well, we may speculate that both labels share patterns. Likely some false positives correspond to the respective other label. Final conclusions are pending more evaluations on larger and more diversified data sets.

Analysis of the stability over time shows the same trends observed in the overall averages. The longitudinal model outperforms both other approaches, while the baseline and IPSS-R models show only small differences for all three metrics and both labels, as seen in Figures A5 and A6.

In conclusion, we determined that our baseline model is well suited for benchmarking our longitudinal model, as it performs similarly to the IPSS-R but is additionally capable of handling missing values and thus allowed us to increase our cohort sizes for training and validation on the complete data set.

### AML Progression as a Prediction Label

We initially qualitatively evaluated AML progression by looking at predictions made by the longitudinal model trained on the 1-year mortality label. As described in the corresponding section, we observed higher risks around the time of AML progression in several patients. Similar to the Comparison With the IPSS-R section above, we also trained our baseline and longitudinal models using the 1-year AML progression risk as the label, but did not limit the data set to just patients with present IPSS-R. Again, we only evaluated the 1-year AML progression label on the Düsseldorf data set using 50 train/test splits. After applying our standard inclusion criteria, n = 831 patients remained with 5,275 samples (3,973 negative samples, without an AML progression 365 days after prediction, and 1,299 positive samples, with AML progression 365 days after prediction). Of the 831 patients, 144 patients had a progression to AML.

As with the 1-year mortality label, we do observe the longitudinal model outperforming the baseline although, with a difference of only 0.1/0.13 for AUROC and AUPRC and a marginally better Brier Score. Although the absolute values are not directly comparable as the data sets are of different sizes and label distributions, we may assume slightly worse overall but still decent performance of both baseline and longitudinal models on this label. All metrics and differences are shown in Table A6.

Once again, performance over time shown in Appendix Figure A7 is the same, sample-length independent performance observed previously. We can conclude that our approach is adaptable to other labels, like the 1-year AML progression label, resulting in the same advantage for the longitudinal model. This again emphasizes the importance of longitudinal data for personalized risk predictions.

**TABLE A1.** Features Extracted From Longitudinal Data

Origin	Feature	Explanation
Tsfresh package^21^	Maximum	See tsfresh package documentation
	Minimum	
	Mean	
	Median	
	Variance	
	standard_deviation	
	variation_coefficient	
	Kurtosis	
	Skewness	
	first_location_of_maximum	
	first_location_of_minimum	
	last_location_of_maximum	
	last_location_of_minimum	
	large_standard_deviation [*r*: 0.05, 0.1, 0.25, 0.5]	
	linear_trend (slope, intercept, *P*)	
	linear_trend_timewise (slope, intercept, *P*)	
	percentage_of_reoccurring_datapoints_to_all_datapoints	
	Quantile [q: 0.1, 0.25, 0.5, 0.75, 0.9]	
	cid_ce [normalize: True]	
Other	Last three points slope	Linear slope of the last three point in the time series. Independent of time

NOTE. Each feature is extracted for each sample and longitudinal parameter for the series of value from diagnosis to the point of prediction.

**TABLE A2.** Hyperparameters for Both Models

Hyperparameter	Baseline Model	Longitudinal Model
n_estimators	500	1,500
learning_rate	0.01	0.01
max_depth	3	3
features_per_split	All	80
random_state	123	123
sample_weight	2.3	2.3

NOTE. Since we use XGBoost, features_per_split are converted to a percentage (features_per_split/total_number_of_features).

**TABLE A3.** Data Set Characteristics for the Different Data Sources

Characteristic	Düsseldorf	Heidelberg	Mannheim
Patients	1,024	286	31
Age, years (diagnosis), median	69	72	72
Follow-up (until event) years, median	2.73	2	4.84
Observation span	1990-2022	2002-2025	2002-2015
Sex			
Female	391	100	18
Male	633	186	13
Mortality during follow-up	658 (64.3%)	167 (58.3%)	12 (38.7%)
AML progression	282 (27.5%)	68 (23.8%)	5 (16.1%)
Samples	6,145	1,708	237
Average samples per patient	6	6	7.6
Label ratio	2.66:1	3:1	10:1
Maximum quarter sampled	32	32	32

NOTE. For data from Heidelberg and Mannheim, no cutoff of the last 60 days was performed. The main objective of the validation data sets is to show performance on real-life data without extensive manual curation and cherry-picking. Still, we see similar median demographics for all data sets with differing label ratios, especially for the Mannheim cohort. The Mannheim cohort has the longest follow-up which is in agreement with the lower proportion of observed mortality events. Follow-up times are given in relation to the mortality event.

**TABLE A4.** Patient and Sample Composition of the Reduced Data Sets for the IPSS-R Comparison

Label	No. of Patients	No. of Samples	No. of Positive Samples	No. of Negative Samples
One-year mortality	688	4,371	1,213	3,158
One-year AML progression	431	3,111	620	2491

NOTE. IPSS-R scores were recalculated from the constituting parameters for all patients. We see a larger label imbalance for the AML progression label since only approximately 30% of patients progress to AML.

**TABLE A5.** AUROC, AUPRC, and Brier Score Averages for the One-Year Mortality and One-Year AML Progression Label

Label	Metric	Baseline	Longitudinal	IPSS-R
One-year mortality	AUROC	0.7037	**0.8098**	0.6784
	AUPRC	0.4871	**0.6181**	0.4646
	Brier score	0.2145	**0.1718**	0.1833
One-year AML progression	AUROC	0.6983	**0.7899**	0.6480
	AUPRC	0.4359	**0.5471**	0.4044
	Brier score	0.2023	**0.1704**	0.2109

NOTE. For AUROC and AUPRC higher, for the Brier Score lower values are better. The longitudinal model performs best for all metrics. Bold entries denote best performing model for the respective metric and dataset.

Abbreviations: AUPRC, area under the precision-recall curve; AUROC, area under the receiver operating characteristic curve.

**TABLE A6.** AUROC, AUPRC, and Brier Score Averages for the One-Year AML Progression Label on the Düsseldorf Data Set

Metric	Baseline	Longitudinal	Difference
AUROC	0.6780	**0.7847**	+0.1067
AUPRC	0.4250	**0.5629**	+0.1379
Brier score	0.2055	**0.1691**	–0.0364

NOTE. For AUROC and AUPRC higher, for the Brier Score lower values are better. The longitudinal model performs best for all metrics. Bold entries denote best performing model for the respective metric and dataset.

Abbreviations: AUPRC, area under the precision-recall curve; AUROC, area under the receiver operating characteristic curve.
